# Transcriptome Analysis Reveals Root Endophyte *Serendipita indica*-Mediated Growth Promotion in Wheat Is Associated with Enhanced Photosynthesis

**DOI:** 10.3390/microorganisms14081665

**Published:** 2026-07-30

**Authors:** Jing Li, Yang Xia, Youwei Xu, Guangjie Han, Chuanming Li, Qin Liu, Lixin Huang, Manman Lin, Nan Zhang, Yurong Lu, Jian Xu

**Affiliations:** 1 Department of Applied Microbiology, National Agricultural Experimental Station for Agricultural Microbiology in Yangzhou, Lixiahe District Institute of Agricultural Sciences in Jiangsu, Yangzhou 225007, China; 20250056@jaas.ac.cn (J.L.); 20212401@jaas.ac.cn (Y.X.); 20122405@jaas.ac.cn (G.H.); 20172401@jaas.ac.cn (C.L.); 19932402@jaas.ac.cn (Q.L.); 20202401@jaas.ac.cn (L.H.); 20240012@jaas.ac.cn (M.L.); 20240021@jaas.ac.cn (N.Z.); 19982402@jaas.ac.cn (Y.L.); 2 College of Plant Protection, Yangzhou University, Yangzhou 225009, China; mz120241523@stu.yzu.edu.cn

**Keywords:** *Serendipita indica*, symbiosis, growth promotion, chlorophyll, wheat

## Abstract

*Serendipita indica* (*S. indica*), a root endophytic fungus of the *Sebacinaceae* family, promotes growth and increases biomass accumulation in a wide range of plant species. However, the mechanism underlying *S. indica*-mediated growth promotion in wheat (*Triticum aestivum* L.), particularly its effects on chlorophyll accumulation, remains poorly understood. In this study, colonization by *S. indica* significantly enhanced shoot growth, biomass accumulation, plant height, fresh weight, dry weight and chlorophyll content in wheat seedlings. Transcriptome analysis revealed extensive transcriptional reprogramming in leaves, characterized by the upregulation of genes involved in chlorophyll biosynthesis and the downregulation of senescence-associated genes. Gene ontology (GO) enrichment analysis indicated that differentially expressed genes (DEGs) were predominantly associated with light-regulated developmental processes, whereas Kyoto Encyclopedia of Genes and Genomes (KEGG) pathway analysis identified significant enrichment in carotenoid biosynthesis and porphyrin metabolism pathways. These transcriptomic results were further validated by reverse transcription quantitative PCR (RT-qPCR), which confirmed the induction of chlorophyll biosynthesis-related genes and the repression of senescence-associated genes following *S. indica* colonization. Collectively, our results indicate that *S. indica* establishes a beneficial association with wheat and promotes chlorophyll accumulation through coordinated regulation of chlorophyll biosynthesis and leaf senescence, which may contribute to enhanced photosynthetic capacity and improved plant growth during the seedling stage.

## 1. Introduction

Microbial symbionts are widely recognized for their role in promoting plant growth by enhancing nutrient acquisition, stimulating development, and protecting against biotic and abiotic stresses [1,2]. *Serendipita indica* (*S. indica*, formerly known as *Piriformospora indica*) is a well-known root endophytic fungus belonging to the order *Sebacinales* [3,4]. Since its first discovery in India in 1997, the beneficial endophytic microorganism has received significant attention in agriculture owing to its distinctive capacity to stimulate plant growth and enhance plant resilience to environmental stressors [5,6,7].

Studies confirmed that *S. indica* formed mutualistic associations with a wide range of monocotyledonous and dicotyledonous plants and significantly enhanced plant growth and yield [8,9,10]. Cereal crops such as wheat, barley, and rice have been identified as hosts of *S. indica* [11,12,13]. *S. indica* colonization in spring barley enhanced plant biomass and increased the grain yield of up to 11% [14]. Seed treatment with *S. indica* significantly promoted vegetative growth and enhanced wheat yield by 16.37% [15]. *S. indica* strongly colonized the roots of Chinese cabbage, promoted root and shoot growth, and promoted lateral root formation [16]. Beyond growth promotion under benign conditions, *S. indica* effectively alleviates plant damage caused by heavy metals, low temperatures, salinity, drought, and pathogens [17,18,19,20]. In addition, the interaction between plants and *S. indica* has been associated with increased phytohormone production, including ethylene (ET), jasmonic acid (JA), gibberellin (GA), salicylic acid (SA), and abscisic acid (ABA). These hormones play critical roles in plant stress responses and development [6,21,22,23,24,25]. To date, however, functional studies on *S. indica* have predominantly centered on its stress tolerance and disease resistance mechanisms. Although the growth-promoting effects of the root endophyte *S. indica* are widely recognized, the underlying mechanisms through which these beneficial effects are achieved remain largely unclear.

Photosynthesis is the fundamental engine driving biomass accumulation and grain yield in plants [26]. Central to this process is the biosynthesis of chlorophyll, the primary pigment responsible for light energy absorption and its subsequent conversion into chemical energy. An increase in chlorophyll content directly enhances the light-harvesting capacity of the thylakoid membrane, thereby optimizing the net photosynthetic rate and fostering plant growth [27]. Beyond pigment concentration, maintaining optimal photosynthetic efficiency strictly relies on the structural integrity and transcriptional regulation of the photosynthetic apparatus. The PsbR protein, an essential component of the Photosystem II (PSII) complex, is crucial for the stable assembly of the oxygen-evolving complex and efficient electron transport [28,29]. Simultaneously, the regulation of leaf greening and pigment homeostasis is often orchestrated by specific transcription factors, such as RADIALIS-Like 3 (RL3), which has been implicated in modulating the chlorophyll degradation pathway [30].

Earlier studies also confirmed that *S. indica* colonization enhanced chlorophyll synthesis and photosynthesis, as well as significantly increased plant growth and yield [31,32]. *S. indica*-colonized barley plants exhibit accelerated development and elevated pho-tosynthetic activity even under light-deficient conditions [14]. Similarly, symbiosis with *S. indica* significantly enhanced the net photosynthetic rate and chlorophyll SPAD values in peanut leaves by 24.17% and 19.66%, respectively [33]. Sweet potato plants colonized by *S. indica* exhibited higher photosynthetic pigment content and improved photosynthesis efficiency compared to non-colonized plants [34]. Although these physiological observations broadly recognize the beneficial impact of *S. indica* on host photosynthetic capacity, the underlying molecular targets and transcriptional regulatory networks remain largely elusive. Therefore, identifying the molecular targets underlying this process is critical for deciphering how *S. indica* modulates the host’s light-harvesting machinery and contributes to improved plant productivity.

Wheat (*Triticum aestivum* L.) is one of the three most important grain species that serves as a staple food for a vast proportion of the world’s population [35]. Due to its ability to produce high yields under a wide range of conditions, wheat is widely cultivated on more land area than any other commercial crop and continues to be the most important food grain source for humans [36]. Fertilizers are widely used to improve wheat yields to meet the increasing food demand. However, the excessive and irrational application of chemical fertilizers has led to soil degradation and environmental pollution, posing a serious threat to the steady and sustainable increase of wheat yields [37]. Therefore, it is imperative to develop and adopt alternative agricultural practices that are environmentally sustainable and can mitigate further environmental degradation. In addition, photosynthesis is a major determinant of wheat growth, biomass accumulation, and yield formation [38]. Because chlorophyll metabolism dictates photosynthetic capacity, clarifying its regulation by *S. indica* is key to unraveling fungal-mediated growth promotion in wheat. Nevertheless, the molecular basis underlying the regulation of wheat chlorophyll metabolism by *S. indica* remains poorly understood.

In this study, we investigated the symbiotic interaction between *S. indica* and wheat through growth phenotyping and transcriptome analysis. Our results demonstrated that *S. indica* colonization significantly promoted wheat growth and was associated with the regulation of genes involved in chlorophyll metabolism. This study provides insights into the early-stage mechanisms of *S. indica*-mediated growth promotion in wheat and contributes to a better understanding of the role of root endophytes in enhancing crop performance.

## 2. Materials and Methods

### 2.1. Plant Material and Fungal Culture

*Serendipita indica* (PI-020) used in this study was isolated by our laboratory from Yangzhou. The isolate was identified based on morphological characteristics and molecular analyses of ITS rDNA and *elongation factor 1-alpha* (*ef1α*) sequences. Phylogenetic analyses confirmed that the isolate belonged to *S. indica*. The isolation and identification procedures were described previously [39].

The wheat seeds (Yangfumai 5) were bred by the Lixiahe Institute of Agricultural Sciences, Yangzhou, China. *S. indica* was cultured on plates of PDA medium (200 g/L potato, 20 g/L dextrose, and 15 g/L agar) at 25 °C for 15 days. The mycelia were scraped and washed with 0.02% tween-80 solution to prepare chlamydospore suspensions of *S. indica*. The suspension was fully mixed using a vortex oscillator for several minutes and diluted with sterilized water to adjust the chlamydospore concentration to 1 × 10^5^/mL [40].

Two identical plastic drums (30 cm in diameter and 35 cm in heigh) were filled with cultivated soil collected from a field and used for wheat cultivation. Wheat seeds were soaked into 75% ethyl alcohol for 5 min and washed with distilled water, then sown in the soil with 30 seeds for each drum, irrigated with 50 mL sterilized water, and cultured in incubator. When the wheats seedling at two leaves stage, 50 mL *S. indica* chlamydospore concentration was irrigated to the surface of the soil of one drum, while the other drum was irrigated with same amount distilled water. After 3 weeks, the seedlings were sampled for the following experiments.

### 2.2. Microscopic Observation and Molecular Detection of S. indica Colonization

To confirm whether *S. indica* had successfully established a symbiotic relationship with wheat, the roots were analyzed by microscopic observation and RT-qPCR. Root samples were randomly collected from wheat seedlings, cut into 0.5-cm segments, and thoroughly washed with sterile water. For microscopic examination, the root segments were treated with 10% KOH for 2 h and then washed with sterilized water. Under a dissecting microscope, the root layers were striped, and the samples were stained with 0.05% trypan blue for 4 min. The stained samples were subsequently observed and documented using a Nikon ECLIPSE NI-U research microscope (Nikon, Tokyo, Japan) [41].

For molecular detection, primers were designed using the NCBI Primer-BLAST tool (https://blast.ncbi.nlm.nih.gov/Blast.cgi, accessed on 2 May 2026) to amplify a fragment of the *S. indica Tef* gene encoding translation elongation factor 1α (GenBank accession number AJ249911.2; Si-forward: 5′-TCCGTCGCGCACCATT-3′ and Si-reverse: 5′-AAATCGCCCTCTTTCCACAA-3′, 84 bp) [42]. The genomic DNA of *S. indica* was used as the template for PCR amplification, and the resulting amplicon was cloned into the pCE2 TA/Blunt-Zero vector (Vazyme, Nanjing, China) to generate the recombinant plasmid pCE2-Si. The recombinant plasmid was used as the standard for absolute quantification by qPCR. A standard curve was generated using a series of ten-fold serial dilutions of the pCE2-Si plasmid.

### 2.3. Determination of Biomass Parameters

Plant height, leaf length, and the fresh weights of roots, stems, and leaves of *S. indica*-colonized and uncolonized wheat plants were determined 20 days after inoculation. Chlorophyll content was measured using a SPAD-502 Plus chlorophyll meter (Konica Minolta Sensing, Inc., Osaka, Japan) [43]. Ten biological replicates were analyzed for each treatment.

### 2.4. Transcriptome Sequencing

Similar amounts of leaf and root tissues from 10 plants at the seedling stage were pooled for each treatment as biological replicates, and three biological replicates were used for transcriptomic analyses. Total RNA was extracted from different samples using the RNA extraction Kit (TaKaRa, Shiga, Japan) according to the manufacturer’s instructions. The purity and integrity of all RNA samples were assessed using a NanoDrop 2000 spectrophotometer (Thermo Fisher Scientific Inc., Waltham, MA, USA). Only samples with an RNA integrity number (RIN) greater than 7.5 were used for sequencing. Approximately 3 µg of RNA per sample was used for cDNA library construction using the Illumina library preparation kit (Illumina, Inc., San Diego, CA, USA) according to the manufacturer’s instructions. The libraries were sequenced on an Illumina HiSeq 2000 platform to generate 100-bp single-end reads (Illumina, Inc., San Diego, CA, USA). Prior to assembly, the qualities of raw data were assessed, and low-quality reads, adapter contamination, and high content of unknown bases were removed to obtain clean reads. The clean reads were aligned to the wheat reference genome (*Triticum aestivum*; NCBI.GCF_018294505.1_IWGSC_CS_RefSeq_v2.1) using HISAT2(v2.0.4) [44], followed by transcript assembly and expression quantification with StringTie(v2.2.1) [45]. Differentially expressed genes (DEGs) were identified using the criteria of |log_2_ (Fold Change)| ≥ 1 and a false discovery rate (FDR) < 0.05.

### 2.5. RNA Extraction and RT-qPCR

One microgram of total RNA was reverse transcribed into cDNA using the PrimeScript RT Reagent Kit (TaKaRa, Shiga, Japan) according to the manufacturer’s instructions. The resulting cDNA was used as the template for gene expression analysis, with *TaACTIN* (*TraesCS1B03G0789600*) serving as the reference gene. The qPCR reaction was performed using AceQ qPCR SYBR Green Master Mix (Vazyme, Nanjing, China) in conjunction with the ABI Prism 7500 real-time PCR system (Applied Biosystems, Foster City, CA, USA). Relative gene expression levels were calculated using the 2^−ΔΔCt^ method [46]. Three technical replicates were included for each sample. Primers used for RT-qPCR for each gene are listed in Supplementary Table S1.

### 2.6. Statistical Analysis

Statistical analysis was conducted in Microsoft Excel via two-tailed Student’s *t*-test. Venn diagram analysis was conducted via the web tool http://bioinfogp.cnb.csic.es/tools/venny/ (accessed on 20 March 2026). GO and KEGG pathway enrichment analyses were performed using the Dr. TOM platform (http://report.bgi.com, accessed on 22 March 2026), an in-house data-mining and visualization system developed by Beijing Genomic Institute (BGI).

## 3. Results

### 3.1. Detection of S. indica Colonization in the Wheat Roots

**Figure 1:**
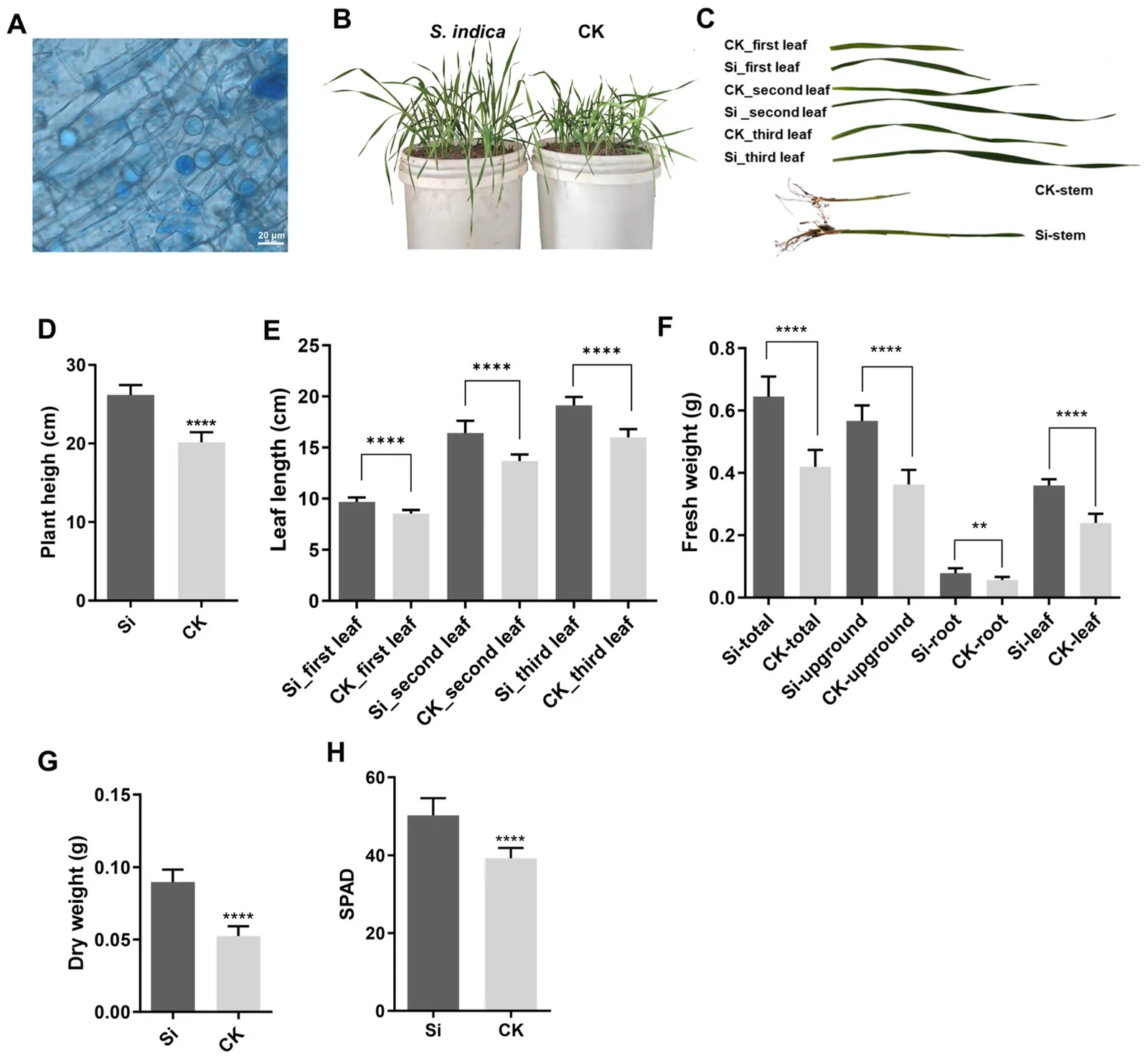
Colonization of *S. indica* in wheat roots and its effects on wheat growth. (**A**) Root colonization by *S. indica* observed under 40× magnification after staining with lactophenol cotton blue. Bar = 20 μm. (**B**) Phenotypes of wheat plants colonized by *S. indica* and non-colonized control plants at 21 days after inoculation. (**C**) Shoots of control and *S. indica* colonized plants. (**D**–**G**) Determination of plant height (**D**), leaf length (**E**), fresh weight (**F**) and dry weight (**G**) in wheat plants colonized with *S. indica* and non-colonized wheat plants. (**H**) Chlorophyll content of wheat seedlings colonized by *S. indica.* Data are shown as means ± standard deviation (SD) (n = 30). Asterisks indicate significant differences from control (**** *p* < 0.0001; ** *p* < 0.01; Student’s *t*-test). Si, wheat plants colonized with *S. indica*; CK, wheat plants without *S. indica* inoculation (control).

To investigate the growth-promoting effects of *S. indica* on wheat, we first established a symbiotic system. Wheat seedlings at the two-leaf stage were inoculated with the fungus via root drenching, and fungal colonization was assessed three weeks after inoculation. Trypan blue staining confirmed the successful colonization of *S. indica* within the root cortical cells, as observed under microscopic inspection (Figure 1A). This symbiotic association was further validated by RT-qPCR analysis. The molecular results revealed a robust accumulation of *S. indica* transcripts in inoculated roots, whereas no fungal transcripts were detected in the uninoculated controls (Supplementary Figure S1). Taken together, these morphological and molecular lines of evidence demonstrated the successful establishment of a symbiotic association between *S. indica* and wheat.

### 3.2. S. indica Enhances Wheat Growth and Chlorophyll Accumulation

Under similar growth conditions, *S. indica* colonization significantly improved wheat growth parameters (Figure 1). At 21 days post-inoculation (dpi), inoculated plants exhibited substantial phenotypic improvements compared with the uncolonized controls. Specifically, plant height increased by 29.91%, while the lengths of the first, second, and third leaves increased by 13.36%, 19.88%, and 19.59%, respectively (Figure 1B–E). Furthermore, biomass measurements showed that total fresh weight increased by 53.65%, accompanied by increases of 56.02% and 38.39% in shoot and root fresh weights, respectively (Figure 1F). The total dry weight of *S. indica*-colonized wheat seedlings was also increased by 56.72% compared with the control plants (Figure 1G). Additionally, the total chlorophyll content increased by 21.88% in colonized plants relative to the control plants (Figure 1H). Collectively, these results demonstrated that *S. indica* colonization promotes wheat seedling growth, accompanied by increased biomass accumulation and enhanced chlorophyll content.

### 3.3. Transcriptome Analysis and Identification of DEGs Induced by S. indica

To investigate the molecular mechanisms underlying *S. indica*-mediated growth promotion in wheat, transcriptome analysis was performed. Twelve leaf and root samples collected from *S. indica*-colonized and control plants were sequenced using the DNBSEQ platform. The sequencing generated between 46.78 and 48.93 million raw reads per sample. After removing low-quality reads and contaminants, the number of clean reads ranged from 44.75 to 45.39 million per sample (Supplementary Table S2). The average mapping rate to the reference genome was 94.04%. In total, 86,878 genes were annotated from the clean reads, and more than 94% of the reads were successfully mapped to protein-coding genes in all samples.

**Figure 2:**
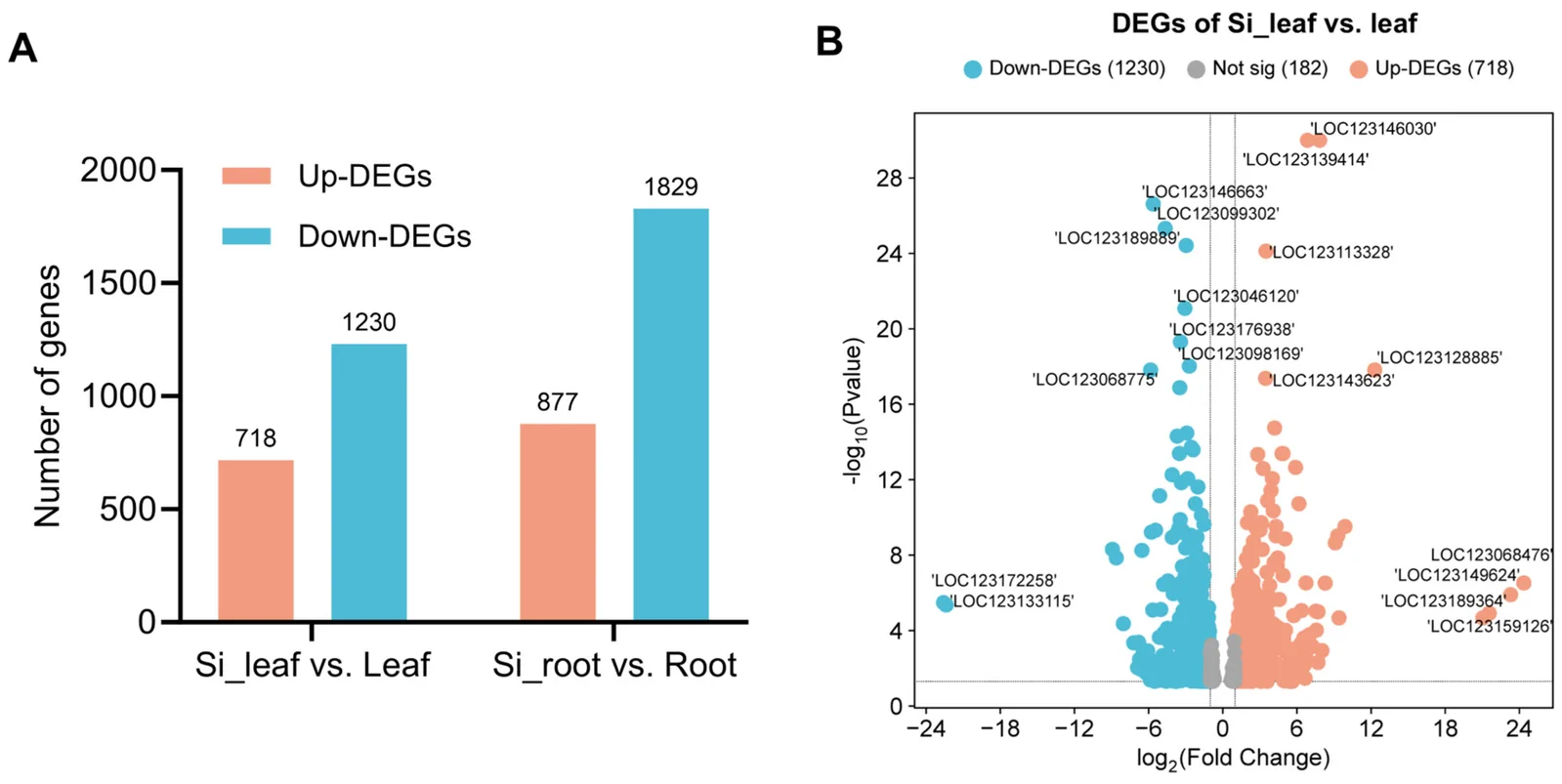
Identification of differentially expressed genes (DEGs) in wheat leaves and roots following *S. indica* colonization. (**A**) Number of DEGs identified in the leaves and roots of wheat plants colonized with *S. indica* and non-colonized wheat plants. DEGs were defined as genes with a fold change (FC) ≥ 2. (**B**) Volcano plot showing upregulated DEGs (orange dots), non-DEGs (gray dots), and downregulated DEGs (blue dots) in the leaves of the wheat plants colonized with *S. indica* and non-colonized wheat plants. The x axis represents log_2_ (FC), while the y axis represents −log_10_ (*p* value). The vertical dashed lines indicate the fold change thresholds (|log_2_ FC| = 1), while the horizontal dashed line represents the significance threshold (*p* value = 0.05). Genes falling outside the area defined by these dashed lines are considered significantly differentially expressed. The screening criteria for DEGs were |log_2_ FC| ≥ 1 and *p* value < 0.05. Si_leaf, leaves from *S. indica*-colonized plants; Si_root, roots from *S. indica*-colonized plants; leaf, leaves from non-colonized control plants; root, roots from non-colonized control plants.

Differential expression analysis was performed between *S. indica*-colonized and control plants using a threshold of |log_2_ fold change| ≥ 1 and a false discovery rate (FDR) < 0.05. A total of 1948 DEGs were identified in leaves, including 718 upregulated and 1230 downregulated genes, whereas 2706 DEGs were identified in roots, including 877 upregulated and 1829 downregulated genes (Figure 2A). More genes were downregulated than upregulated in both tissues, and the number of downregulated genes was greater in roots than in leaves. Several representative DEGs were highlighted in the volcano plots. Notably, the chlorophyll-related gene *TaPsbR* was significantly upregulated, whereas the senescence-associated gene *TaRL3* was significantly downregulated in leaves (Figure 2B; Table 1). These results suggest that *S. indica* colonization may influence wheat growth through transcriptional regulation of genes associated with chlorophyll biosynthesis and senescence.

### 3.4. Functional Enrichment Analysis of DEGs in Response to S. indica Colonization

**Figure 3:**
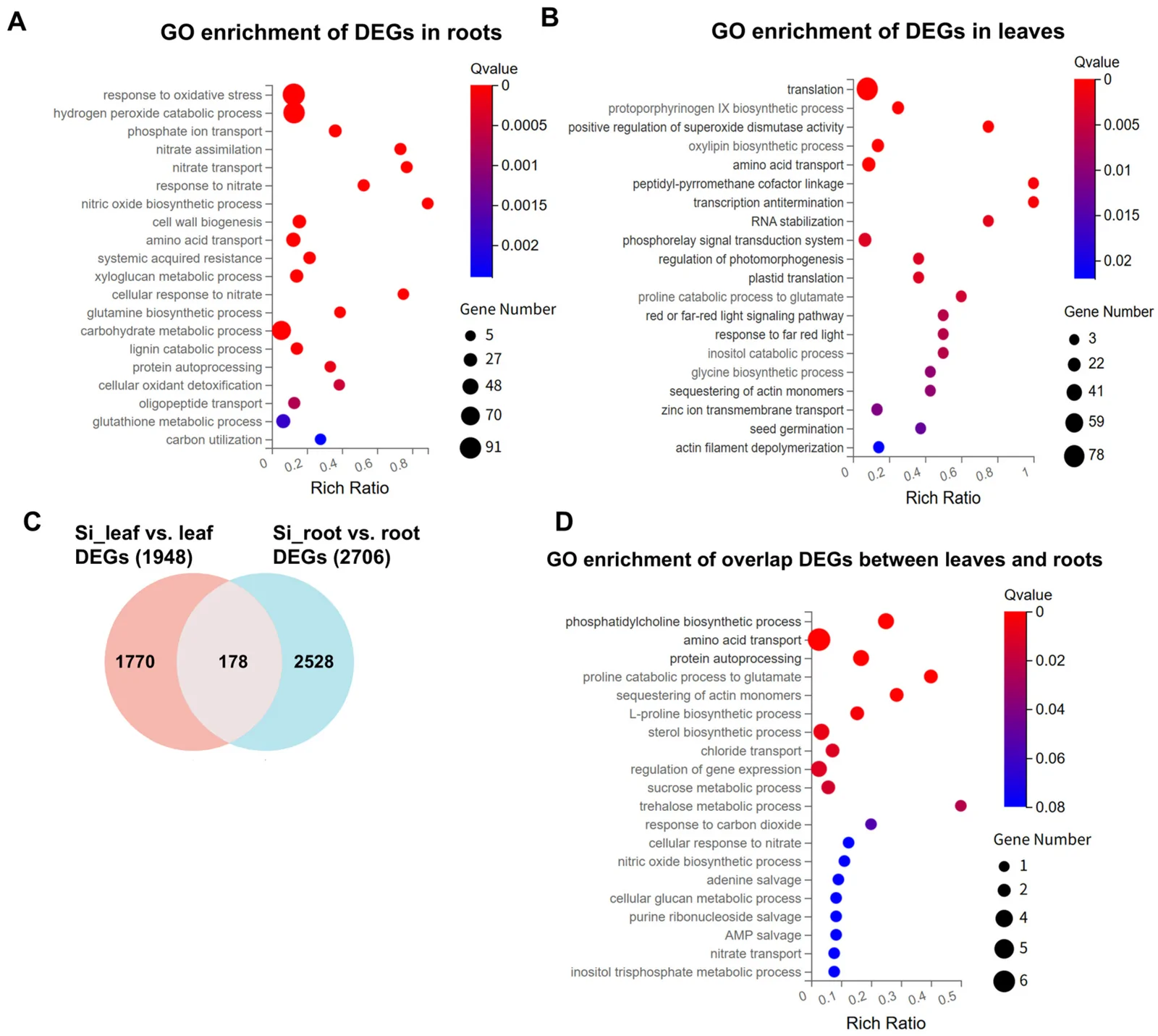
Gene Ontology (GO) enrichment analysis of DEGs in wheat following *S. indica* colonization. (**A**,**B**) Bubble diagram of GO enrichment analysis of DEGs (colonized versus non-colonized) in roots (**A**) and leaves (**B**). (**C**) Venn diagrams showing the overlap between root and leaf DEGs identified in *S. indica*-colonized and non-colonized wheat plants. (**D**) Bubble diagram of GO enrichment analysis of the overlapping DEGs (colonized versus noncolonized) between leaves and roots. The bubble size indicates the number of DEGs, and the color gradient represents the Q-value. Red bubbles indicate lower Q-values and more significant enrichment, whereas blue bubbles indicate higher Q-values and less significant enrichment. Si_leaf, leaves from *S. indica*-colonized plants; Si_root, roots from *S. indica*-colonized plants; leaf, leaves from non-colonized control plants; root, roots from non-colonized control plants.

GO enrichment analysis was performed to characterize the biological functions of the DEGs. In roots, the 2706 DEGs identified following *S. indica* colonization were significantly enriched in GO terms such as “response to oxidative stress”, “hydrogen peroxide catabolic process”, “phosphate ion transport”, “nitrate assimilation”, “nitrate transport” and “lignin catabolic process” (Figure 3A). In leaves, the 1948 DEGs induced by *S. indica* colonization were significantly enriched in GO terms such as “translation”, “protoporphyrinogen IX biosynthetic process”, “positive regulation of superoxide dismutase activity”, “oxylipin biosynthetic process”, “red or far-red light signaling pathway” and “regulation of photomorphogenesis” (Figure 3B). Venn diagram analysis revealed 178 overlapping DEGs between leaves and roots (Figure 3C). These shared DEGs were enriched in GO terms such as “phosphatidylcholine biosynthetic process”, “amino acid transport”, “proline catabolic process to glutamate”, “sucrose metabolic process” and “trehalose metabolic process” (Figure 3D). These results indicated that *S. indica* colonization induces tissue-specific transcriptional responses in wheat, with roots mainly associated with stress and nutrient metabolism, while leaves were characterized by the regulation of chlorophyll biosynthesis and photosynthesis-related pathways.

**Figure 4:**
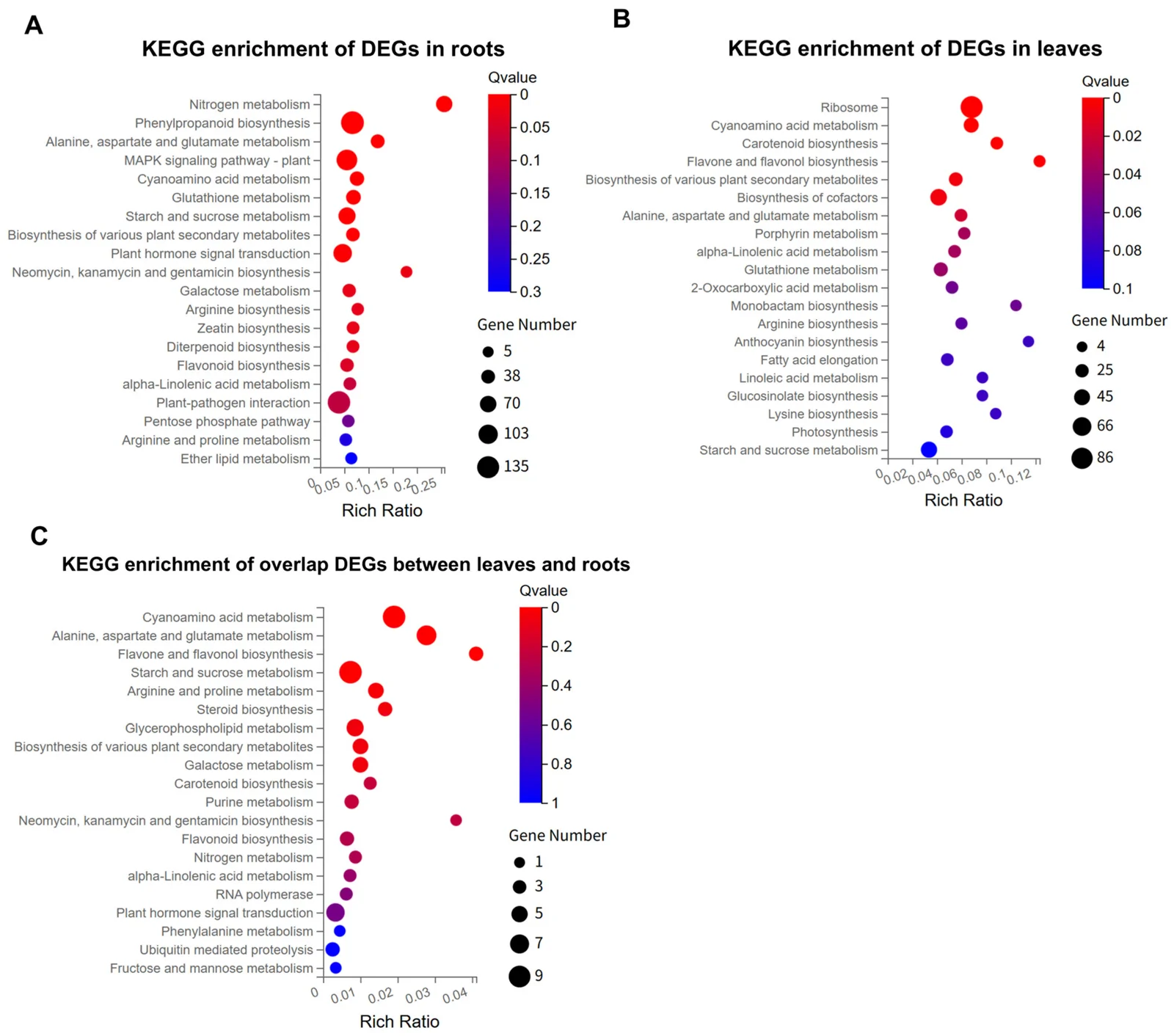
Kyoto Encyclopedia of Genes and Genomes (KEGG) pathway enrichment analysis of DEGs in wheat following *S. indica* colonization. (**A**,**B**) Bubble diagram of KEGG pathway enrichment analysis of DEGs (colonized versus noncolonized) identified in roots (**A**) and leaves (**B**). (**C**) Bubble diagram of KEGG pathway enrichment analysis of the overlapping DEGs (colonized versus noncolonized) between leaves and roots. The bubble size indicates the number of DEGs, and the color gradient represents the Q-value. Red bubbles indicate lower Q-values and more significant enrichment, whereas blue bubbles indicate higher Q-values and less significant enrichment. Si_leaf, leaves from *S. indica*-colonized plants; Si_root, roots from *S. indica*-colonized plants; leaf, leaves from non-colonized control plants; root, roots from non-colonized control plants.

Subsequently, KEGG pathway analysis was performed to identify the metabolic pathways significantly enriched with DEGs. The DEGs in roots induced by *S. indica* colonization were enriched in KEGG pathways such as “nitrogen metabolism”, “phenylpropanoid biosynthesis”, “alanine, aspartate and glutamate metabolism”, “MAPK signaling pathway-plant” and “cyanoamino acid metabolism” (Figure 4A). The DEGs in leaves induced by *S. indica* colonization were enriched in KEGG pathways such as “ribosome”, “carotenoid biosynthesis”, “flavone and flavonol biosynthesis”, “porphyrin metabolism” and “photosynthesis” (Figure 4B). In addition, the overlapping DEGs identified in both leaves and roots were enriched in KEGG pathways such as “cyanoamino acid metabolism”, “alanine, aspartate and glutamate metabolism”, “flavone and flavonol biosynthesis”, “starch and sucrose metabolism” and “arginine and proline metabolism” (Figure 4C).

GO and KEGG enrichment analyses suggested that wheat responded to *S. indica* colonization through transcriptional reprogramming of genes involved in defense responses and energy metabolism, which may facilitate the establishment of symbiosis. In conclusion, these findings indicate that colonization by *S. indica* induces a systemic response in the host that promotes growth, particularly the synthesis of chlorophyll.

### 3.5. Expression of Wheat Chlorophyll Metabolism-Related Genes During S. indica Colonization

**Figure 5:**
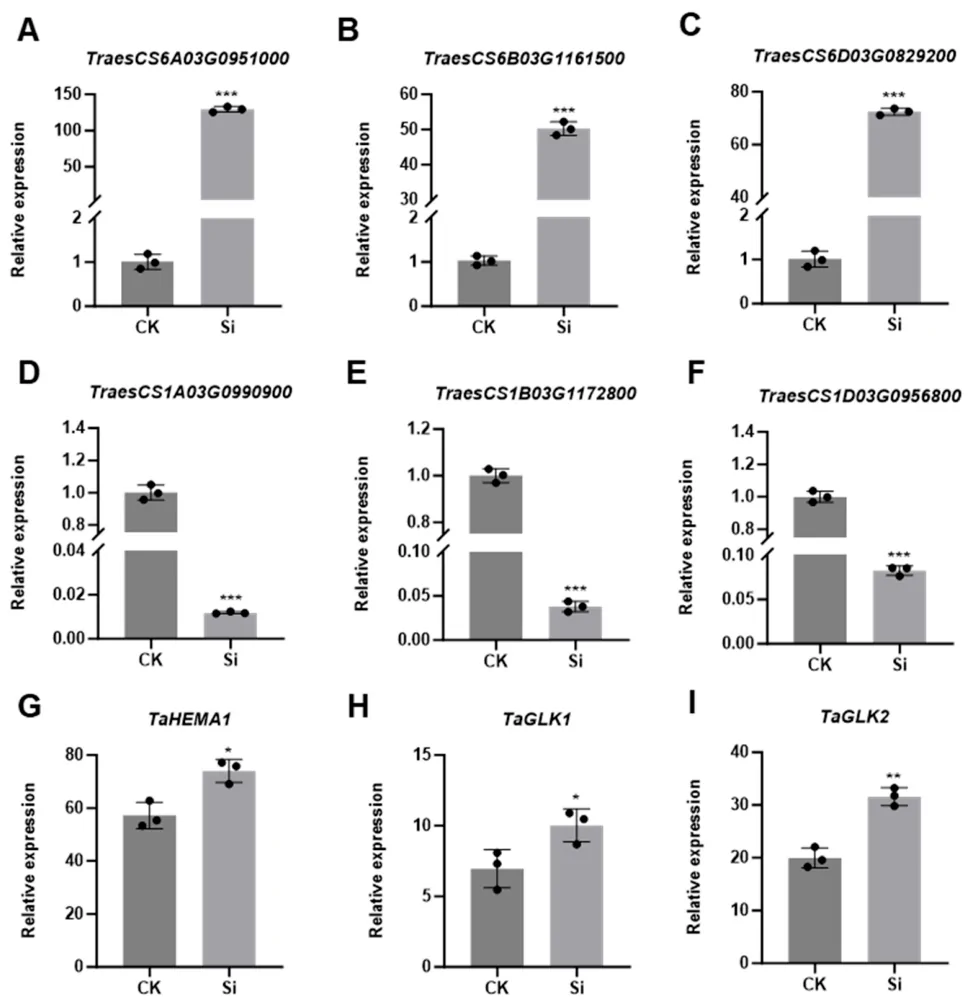
Expression analysis of chlorophyll metabolism-related genes in wheat leaves following *S. indica* colonization. (**A**–**I**) Relative transcript levels of chlorophyll metabolism-related genes were determined by RT-qPCR. Expression levels were normalized by the gene *TaACTIN* whole expression is set as 1. Data are the means of 3 technical replicates and error bars represent SD. Similar results were obtained in 3 independent experiments. Lowercase letters above the bars indicate significant differences among samples at *p* < 0.05, by SSR-test. Asterisks indicate significant differences from control (*** *p* < 0.001; ** *p* < 0.01; * *p* < 0.05; Student’s *t*-test). Si, wheat plants colonized with *S. indica*; CK, wheat plants without *S. indica* inoculation (control).

In view of the fact that chlorophyll metabolism plays a vital role in regulating plant photosynthesis and overall growth and development [47], it is crucial to understand how *S. indica* influences this pathway. We analyzed the relative expression levels of chlorophyll synthesis-related genes in wheat plants during colonization with *S. indica* by RT-qPCR. Gene expression analysis showed that *S. indica* colonization significantly increased the transcript abundance of the three wheat homoeologous copies of *TaPsbR* (*TraesCS6A03G0951000*, *TraesCS6B03G1161500*, and *TraesCS6D03G0829200*) in leaves (Figure 5A–C). In contrast, *S. indica* colonization significantly downregulated the expression of the three *TaRL3* homeologs (*TraesCS1A03G0990900*, *TraesCS1B03G1172800*, and *TraesCS1D03G0956800*) (Figure 5D–F). This demonstrates that post-colonization growth promotion by *S. indica* is achieved through photosynthetic enhancement via upregulated chlorophyll synthesis and mitigated chlorophyll degradation, which is consistent with our observed increase in leaf chlorophyll content. To further characterize the transcriptional regulation of chlorophyll metabolism, the expression levels of several key genes involved in chlorophyll biosynthesis were examined. The relative expression level of the chlorophyll synthesis gene *TaHEMA1* was markedly elevated in colonized plants, while the transcript levels of the upstream transcription factors *TaGLK1* and *TaGLK2* were also increased in *S. indica*-colonized wheat leaves (Figure 5G–I). Collectively, these results suggest that *S. indica* colonization promotes wheat growth by modulating the expression of chlorophyll metabolism-related genes, thereby enhancing chlorophyll accumulation and photosynthetic capacity in host leaves.

## 4. Discussion

Plants harbor a diverse community of endophytic microorganisms that forms mutualistic associations with their hosts through long-standing evolutionary interactions. Among these endophytes, *S. indica* has attracted considerable attention due to its ability to promote plant growth, enhance stress tolerance, and improve resistance to plant diseases [48]. Despite these widely recognized benefits, the interaction between *S. indica* and wheat remains insufficiently understood, particularly regarding its effects on chlorophyll biosynthesis and metabolism. Therefore, the mechanisms by which *S. indica* regulates chlorophyll metabolism to promote wheat growth remain to be elucidated.

In the present study, phenotypic analyses showed that *S. indica* colonization significantly increased plant height, leaf development, and biomass accumulation, accompanied by elevated chlorophyll content in wheat leaves (Figure 1). Transcriptome analysis further revealed that fungal colonization altered the expression of genes associated with chlorophyll metabolism, photosynthesis, and plant developmental processes. In particular, *S. indica* induced the expression of photosynthesis-related genes such as *TaPsbR* while repressing the senescence-associated gene *TaRL3*. GO and KEGG enrichment analyses demonstrated that leaf DEGs were significantly enriched in pathways related to tetrapyrrole biosynthesis, carotenoid biosynthesis, porphyrin metabolism, and photosynthesis (Figure 3 and Figure 4). RT-qPCR analysis of chlorophyll metabolism-related genes also indicated enhanced chlorophyll biosynthesis in leaves (Figure 5). Since tetrapyrroles are essential precursors for chlorophyll biosynthesis and carotenoids are critical components of the photosynthetic apparatus [49], these results indicate that *S. indica* promotes the establishment of an enhanced photosynthetic system in wheat seedlings.

Photosynthesis enables plants to transform solar energy into chemical energy, thereby providing the energy required for growth and development [50,51]. Chlorophyll biosynthesis is tightly regulated by complex transcriptional networks involving nuclear-encoded regulators and chloroplast-associated processes [52]. HEMA1 functions as a key enzyme in the chlorophyll biosynthetic pathway, while GOLDEN2-LIKE (GLK)transcription factors regulate chloroplast development and activate downstream chlorophyll biosynthesis genes [53,54,55]. Consistent with their established functions, *TaHEMA1*, *TaGLK1*, and *TaGLK2* were significantly upregulated following *S. indica* colonization (Figure 5). These results provide molecular evidence that *S. indica* enhances chlorophyll accumulation through activation of chlorophyll biosynthetic pathways. However, the upstream regulatory mechanisms responsible for the activation of these transcriptional networks remain unknown. Future studies should determine whether *S. indica*-derived signals regulate HY5-, GLK-, or other transcription factor-mediated pathways controlling chloroplast development and photosynthetic activity.

In addition to regulating leaf photosynthetic processes, *S. indica* may enhance plant growth by improving nutrient acquisition through root colonization. In this study, root transcriptome analysis revealed significant enrichment of genes associated with nitrogen metabolism, nitrate transport, and nitrate assimilation (Figure 3A and Figure 4A). Nitrogen availability is closely associated with chlorophyll accumulation and photosynthetic capacity, as nitrogen is an essential component of chlorophyll molecules and is extensively invested in photosynthetic proteins, including Rubisco and thylakoid proteins [56]. Previous studies have shown that *S. indica* enhances nutrient acquisition by promoting root development and regulating nutrient-related metabolic pathways [57]. Therefore, improved nitrogen acquisition and utilization mediated by *S. indica* may provide additional metabolic support for chlorophyll accumulation and photosynthetic enhancement in leaves. These findings suggest that fungal colonization coordinates belowground nutrient acquisition with aboveground photosynthetic development, contributing to overall plant growth promotion.

Numerous studies have demonstrated that inoculation with *S. indica* not only promotes biomass accumulation during the seedling and vegetative growth stages but also exerts sustained growth-promoting effects and regulates yield formation during the reproductive stage. *S. indica* colonization accelerated early developmental processes in barley and significantly increased final grain yield [14]. *S. indica* could promote earlier flowering and increase floral bud formation by regulating gibberellin (GA) metabolic pathways and photoperiod-responsive processes [58]. In rice and wheat, field and greenhouse experiments have also demonstrated that *S. indica* inoculation improves yield-related traits, including increased spike number, enhanced thousand-grain weight, and improved final yield production [15,59]. In the present study, we demonstrated that *S. indica* promotes wheat growth at the seedling stage by enhancing chlorophyll accumulation and photosynthesis-related processes. However, because this study was limited to the seedling stage, future studies should evaluate whether the beneficial effects of *S. indica* are maintained throughout the reproductive stage and determine their impact on grain yield, yield components, and grain quality under field conditions.

In natural soil ecosystems, plant rhizospheres harbor highly diverse and complex microbial communities [60]. Increasing evidence has indicated that co-inoculation of *S. indica* with other beneficial rhizosphere microorganisms often results in complementary or synergistic effects on plant growth and stress tolerance. For example, Co-inoculation of *S. indica* with plant growth promoting rhizobacteria (PGPR) and *Mesorhizobium cicer* in chickpea has been shown to exert synergistic effects, significantly enhancing root dry biomass, nutrient acquisition, and plant stress tolerance [61]. Furthermore, co-colonization of rice with *S. indica* and the nitrogen-fixing bacterium *Azotobacter chroococcum* significantly enhanced carbon allocation and improved nitrogen and phosphorus acquisition efficiency [62]. In addition, *S. indica* has been shown to interact with *Enterobacter* sp. SA187 in Arabidopsis, where the coordinated regulation of ethylene and auxin signaling pathways by these two microorganisms markedly promoted plant growth and enhanced salt tolerance [63]. Therefore, further elucidation of the interaction networks between *S. indica* and other PGPR, as well as the underlying mechanisms of their combined effects on crop growth promotion, will be essential for advancing the development of efficient microbial consortia and facilitating the field application of *S. indica*-based bioinoculants.

## 5. Conclusions

Overall, our study demonstrates that *S. indica* colonization significantly enhances wheat seedling growth by coordinating the expression of genes associated with chlorophyll metabolism, chloroplast development, and photosynthesis. Integrating transcriptomic and physiological evidence, we show that *S. indica* promotes chlorophyll accumulation and elevates photosynthetic capacity, thereby driving biomass accumulation. These findings provide new insights into the molecular mechanisms underlying *S. indica*-mediated growth promotion and highlight the importance of chlorophyll metabolic regulation in fungus-plant symbiosis.

## Figures and Tables

**Table 1:** The most significantly expressed genes from the volcano plot in wheat leaves colonized by *S. indica*.

Gene	Gene	Annotation
*LOC123068476*	*TraesCS3B03G0142900*	non-specific lipid-transfer protein Cw18
*LOC123149624*	*-*	putative receptor-like protein kinase At4g00960
*LOC123189364*	*TraesCS2A03G0787500*	uncharacterized LOC123189364
*LOC123159126*	*-*	DIMBOA UDP-glucosyltransferase BX8-like
*LOC123143623*	*TraesCS6D03G0183200*	chloride channel protein CLC-b
*LOC123128885*	*TraesCS6A03G0951000*	**photosystem II 10 kDa polypeptide, PsbR**
*LOC123113328*	*TraesCS5B03G0981700*	beta-glucosidase BoGH3B
*LOC123146030*	*TraesCS6D03G0829200*	**photosystem II 10 kDa polypeptide, PsbR**
*LOC123139414*	*TraesCS6B03G1161500*	**photosystem II 10 kDa polypeptide, PsbR**
*LOC123133115*	*TraesCS6B03G0035400*	BURP domain-containing protein 3
*LOC123172258*	*TraesCSU03G0019400*	sugar transporter ERD6-like 5
*LOC123068775*	*TraesCS1A03G0990900*	**protein RADIALIS-like 3, RL3**
*LOC123098169*	*TraesCS4D03G0572200*	branched-chain-amino-acid aminotransferase 2, chloroplastic
*LOC123176938*	*TraesCS1D03G0956800*	**protein RADIALIS-like 3, RL3**
*LOC123046120*	*TraesCS2B03G1018400*	probable low-specificity L-threonine aldolase 1
*LOC123189889*	*TraesCS2A03G0926300*	probable low-specificity L-threonine aldolase 1
*LOC123099302*	*TraesCS1B03G1172800*	**protein RADIALIS-like 3, RL3**
*LOC123146663*	*TraesCS1B03G1174300*	**protein RADIALIS-like 3, RL3**

## Data Availability

The data presented in this study are openly available in NCBI Sequence Read Archive, BioProject PRJNA1481803.

## Supplementary Materials

The following supporting information can be downloaded at: https://www.mdpi.com/article/10.3390/microorganisms14081665/s1, Figure S1: Detection of *S. indica* colonization in wheat roots. (A) Standard curve generated from ten-fold serial dilutions of the recombinant plasmid pCE2-Si for the absolute quantification of *S. indica* colonization in wheat roots (R^2^ = 0.9969). (B) Absolute colonization abundance of *S. indica* in wheat roots determined by qPCR. Si-1, Si-2, and Si-3 represent three independent wheat root samples. Table S1: Primers used in this study. Table S2: RNA-seq sequencing information.
