# Supplementary material for: Transcriptome Analysis Reveals Root Endophyte Serendipita indica-Mediated Growth Promotion in Wheat Is Associated with Enhanced Photosynthesis
Source: Microorganisms. 2026 Jul 30;14(8):1665. doi: 10.3390/microorganisms14081665 (PMC13515818; doi:10.3390/microorganisms14081665)
Supplement: Supplementary file 1 [file microorganisms-14-01665-s001.zip › microorganisms-4430372-supplementary-tables.pdf]

Supplementary Table S1. Primers used in this study.

Primers	Primer sequence	Purpose
TaACTIN1-qRT-F	ACCTTCAGTTGCCCAGCAAT	RT-qPCR
TaACTIN1-qRT-R	CAGAGTCGAGCACAATACCAGTTG	RT-qPCR
TraesCS6A03G0951000-qRT-F	GTTGATGGGTACAGCCCGAT	RT-qPCR
TraesCS6A03G0951000-qRT-R	TTCCAGCAAGAGTCACTGCC	RT-qPCR
TraesCS6B03G1161500-qRT-F	TTTCACTGTTGTGCATCGCT	RT-qPCR
TraesCS6B03G1161500-qRT-R	CAGCCCTGTCTTTCCTCCAA	RT-qPCR
TraesCS6D03G0829200-qRT-F	GAAACTCCGAGGTTTGCGAC	RT-qPCR
TraesCS6D03G0829200-qRT-R	GTAGACACCCTTCCCCTTGG	RT-qPCR
TraesCS1A03G0990900-qRT-F	CGTCAACAACATCGAGTACGG	RT-qPCR
TraesCS1A03G0990900-qRT-R	GAGTTTACACTGCTTCATCCAAT	RT-qPCR
TraesCS1B03G1172800-qRT-F	GTATGGCCGCATCCCCTTC	RT-qPCR
TraesCS1B03G1172800-qRT-R	GGAGTTTACACTGCTTCATCCTTTC	RT-qPCR
TraesCS1D03G0956800-qRT-F	TCTGGACCTGAAAGGATAAAGC	RT-qPCR
TraesCS1D03G0956800-qRT-R	TGCTGGATTGCTTGCTTCCTA	RT-qPCR
TaHEMA1-qRT-F	AGATCTCTGCGGACCGGTAT	RT-qPCR
TaHEMA1-qRT-R	GTCCACTGGTGCTGTGTGTA	RT-qPCR
TaGLK1-qRT-F	CGTGTCCAGTCTACACGCC	RT-qPCR
TaGLK1-qRT-R	TGCTCTCGTTTGATGGTTGTG	RT-qPCR
TaGLK2-qRT-F	CGGGTCTGAGAACAGCAAGT	RT-qPCR
TaGLK2-qRT-R	GTCCAGTCCACCTTCCGC	RT-qPCR

Supplementary Table S2. RNA-seq sequencing information.

Sample	Total Raw Reads (M)	Total Clean Reads (M)	Total Mapping(%)
Leaf1	48.93	45.39	95.41
Leaf2	47.19	44.89	96.35
Leaf3	47.19	45.19	96.52
Si_leaf1	47.19	45.03	96.46
Si_leaf2	47.19	44.32	96.32
Si_leaf3	47.19	44.92	96.56
Si_root1	47.19	44.76	94.43
Si_root2	47.19	44.09	95.11
Si_root3	47.19	45.17	96.39
Root1	47.19	45.05	92.82
Root2	46.78	44.75	93.87
Root3	47.19	44.77	78.27
