# Supplementary material for: Transcriptome Analysis Reveals Root Endophyte Serendipita indica-Mediated Growth Promotion in Wheat Is Associated with Enhanced Photosynthesis
Source: Microorganisms. 2026 Jul 30;14(8):1665. doi: 10.3390/microorganisms14081665 (PMC13515818; doi:10.3390/microorganisms14081665)
Supplement: Supplementary file 1 [file microorganisms-14-01665-s001.zip › microorganisms-4430372-supplementary-figures.pdf]

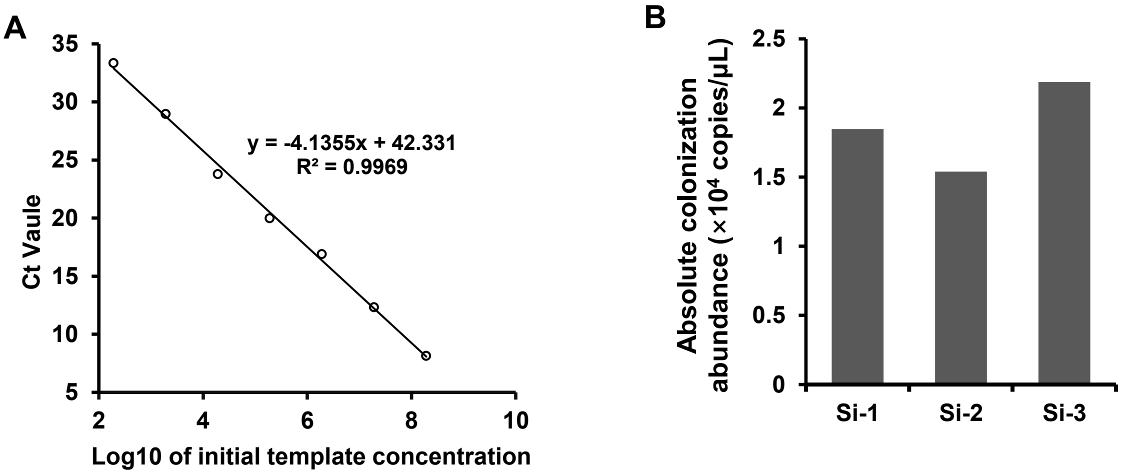

Supplementary Fig. S1. Detection of *S. indica* colonization in wheat roots. **(A)** Standard curve generated from ten-fold serial dilutions of the recombinant plasmid pCE2-Si for the absolute quantification of *S. indica* colonization in wheat roots ($R^2 = 0.9969$). (**B**) Absolute colonization abundance of *S. indica* in wheat roots determined by qPCR. Si-1, Si-2, and Si-3 represent three independent wheat root samples.
